# Association of Residence Type on Smoking in South Korean Adolescents during the COVID-19 Pandemic: Findings from a National Survey

**DOI:** 10.3390/ijerph191912886

**Published:** 2022-10-08

**Authors:** Mi Young Kwon, Myoung Sun Cho

**Affiliations:** 1 College of Nursing, Ewha Womans University, Seoul 03760, Korea; 2 Department of Nursing, Gangneung-Wonju National University, Wonju 26403, Korea

**Keywords:** adolescent health, smoking, tobacco use, residence characteristics

## Abstract

The closure of schools due to the COVID-19 pandemic has forced adolescents to stay home. These disruptions, as well as a significant decrease in social access, have impacted smoking behavior. This study identified the association between the adolescents’ type of residence and tobacco product use. A cross-sectional study (using data from the Korea Youth Risk Behavior Web-based Survey) examined 3774 students in 2019 (pre-pandemic) and 2575 students in 2020 (during the pandemic). The participants were South Korean middle and high school students aged 13–19 years. Using multinomial logistic regression, it was shown that adolescents who lived alone or in a boarding house had a higher risk of being an e-cigarette smoker compared with those who lived with family or relatives (OR = 6.49, CI = 2.06–20.45). Living in a dormitory or orphanage also increased the risk of dual tobacco use compared with living with family (OR = 2.09, 95% CI = 1.13–3.84). With the advent and continuation of the COVID-19 pandemic, this effect became more significant in 2020 than in 2019. Our findings support the theory that residential differences affect adolescent smoking behavior and highlight the importance of integrated smoking bans and educational programs to control adolescent smoking.

## 1. Introduction

As smoking behaviors that begin in adolescence have a high risk of leading to lifelong smoking, it is important to continuously monitor the smoking status of adolescents [1]. With the advent of odorless e-cigarettes with different flavors and designs based on new technologies, the types of tobacco used by all smokers [2,3] and adolescent smokers are changing [4]. According to a study analyzing trends in the types of tobacco used by adolescents, the number of adolescents who use cigarettes worldwide has been steadily declining; however, the number of adolescents using e-cigarettes is constantly increasing [5]. This changing trend in the type of cigarette indicates the necessity to change policy efforts and interventions, such as the control of cigarette prices and sales, the anti-smoking campaign, and the establishment of non-smoking zones that traditionally focused on cigarettes [2,3].

Adolescents who use e-cigarettes tend to pursue sensations and individuals who use psychologically active substances are more likely to use one or more substances, so there is a high risk of using substances such as alcohol and drugs [6]. Moreover, various substances contained in e-cigarette liquids can adversely affect adolescents’ health [7]. Unlike cigarettes, which can be purchased primarily through retail stores, e-cigarettes can also be purchased at dedicated offline retailers and online sales sites. In addition, e-cigarette store advertisements, which emphasize attractive designs and flavors that differ from conventional cigarettes, increase the intent of use for both youth smokers and non-smokers [7,8]. The emergence of new types of tobacco has given rise to new knowledge and perceptions about smoking [9]. In a long-term follow-up of a panel of adult smokers who used cigarettes in Korea, the most common reasons for the use of e-cigarettes was that they were less likely to be harmful to health, did not smell, and could be smoked secretly indoors in a non-smoking place because there is no smell and smoke [10]. Although all types of tobacco contain a variety of harmful substances, including nicotine [11], many adolescents consider e-cigarettes to be less harmful and addictive to the human body than cigarettes; therefore, they often use e-cigarettes as a substitute for cigarettes [1]. In the early days of the advent of e-cigarettes, the use of these was expected to help cigarette smokers quit smoking successfully [7]. However, contrary to expectations, the absence of visible smoke and odor has led to the emergence of adolescent dual users who switch to e-cigarettes that are free from the restrictions of non-smoking zones or those who use cigarettes and e-cigarettes in combination [4,12]. With an increase in adolescent dual users in Korea, it is insufficient to understand adolescent smoking by categorizing it as e-cigarette or regular cigarette consumption [13].

The type of tobacco used by adolescents is influenced by a variety of environmental and personal factors, such as stress, drug use, the age at which they start smoking, and their family’s economic level [14,15]. A study on the environmental factors influencing smoking defined sudden changes in the environment, such as changes in the family and limited resources, as “context effects”; the COVID-19 pandemic can also be seen as a context effect that impacted adolescent smoking behavior [16]. The need for belonging to a peer group, the need for attention, surveillance by parents or family members, and family features such as family members’ smoking is known to be related not only to adolescent smoking behavior but also to the type of tobacco used [1,17,18]. However, the COVID-19 pandemic has led to changes in these factors and their effects on smoking [8]. Shutdowns and social distancing have made it difficult for adolescents to meet with their peers; they have spent more time at home with temporary school interruptions or repeated school closures [8,19].

One of the significant environmental factors influencing adolescents’ smoking behavior is the type of residence, including who they cohabit with, their relationship with their cohabitants, and the physical type of living space [8,18,20,21]. Adolescents who do not live with their parents or family members have a higher risk of smoking due to a lack of care and surveillance by guardians, and those who live with family members have a higher risk of smoking if someone smokes at home [8,22,23]. Cohabitation with family members or relatives also lowers adolescents’ risk of smoking because it provides stability in the family structure due to the presence of a primary guardian [21]. In addition, when sharing a living space, such as cohabitation with others or living in a group residential facility, there are formal or informal regulations for the non-smoking zone, which affects the type of tobacco which is used [4,24,25].

In 2019, COVID-19 spread worldwide after it was discovered with pneumonia of an unknown cause, followed by the World Health Organization declaring a pandemic, the highest level of infectious disease risk. Given the restrictions imposed to prevent the spread of infection in the early stages of the pandemic, the use of e-cigarettes among adolescents was expected to decrease; however, this trend continues to increase [7]. According to the Korea Disease Control and Prevention Agency report, the smoking rate of adolescents in 2021 was 6.0% in males and 2.9% in females, similar to that of 2020, but the use of liquid e-cigarettes increased by 1.0% in males (from 2.7 percent to 3.7%) and 0.8% in females (from 1.1% to 1.9%) [26]. Since the outbreak of COVID-19, studies on the types of tobacco used and residence of adolescent smokers have focused primarily on whether they cohabitate with parents or family members [8,19]. However, the type of residence varies widely [24]. Considering the relevance of the type of residence to the type of tobacco used [24,25,27], it is necessary to understand the relationship between these two factors in the context of the COVID-19 pandemic. Accordingly, this study compared pre- and post-COVID-19 periods to understand the contextual characteristics of the pandemic and the impact of the residence type of adolescent smokers on the type of tobacco used.

## 2. Materials and Methods

### 2.1. Data, Design, and Participants

This cross-sectional study analyzed data from the Korea Youth Risk Behavior Web-Based Survey (KYRWBS) in 2019 and 2020 to examine the impact of the type of residence on the type of tobacco used by adolescents. The KYRWBS is a national survey conducted annually in South Korea to understand the status and trends of adolescents’ health behaviors. Participants were selected from 400 high schools across the country using a stratified two-stage cluster sampling method. In Stage 1 sampling, the sample schools were selected by systematic sampling based on a list of schools that comprised the main population, followed by Stage 2 sampling, in which one class per grade was randomly selected per sampled school. The survey was conducted anonymously online.

This study included survey data from June 2019 and from August to November 2020 to compare the pre- and post-COVID-19 periods. There were 57,303 participants in the 2019 survey and 54,948 in 2020. Adolescents who responded that they had smoked within the last month to the question regarding whether they smoked were included in the study, and then classified according to the type of tobacco they smoked. First, those who reported only smoking cigarettes were classified as “cigarette users,” and those who reported smoking only e-cigarettes were classified as “e-cigarette users”; participants who reported smoking using both cigarettes and e-cigarettes were classified as “dual users.”

In 2019, there were a total of 3774 adolescent smokers, comprising 1817 cigarette users (47.2%), 298 e-cigarette users (8.2%), and 1659 dual users (44.6%). In 2020, there were 2575 adolescent smokers, including 1407 cigarette users (54.5%), 205 e-cigarette users (8.8%), and 963 dual users (36.6%).

### 2.2. Variables

The independent variable in this study was the type of residence. The survey respondents were asked “What is your current type of residence?”. The responses were as follows: living with family, living in a relative’s house, living in a boarding house or living alone, living in a dormitory, and living in childcare facilities (orphanages, social welfare facilities, or nursery schools). These data were categorized into: (1) living with family or relatives, (2) living alone or in a boarding house, and (3) living in a dormitory or orphanage.

The dependent variable was the type of tobacco used, which was classified as cigarettes, e-cigarettes, and mixed (dual) use, according to the type used in the last 30 days.

In this study, variables known to affect adolescents’ choice of tobacco use were used as correction variables to determine the relationship between the dependent and independent variables. The correction variables were: sex; age; perceived family’s economic status; stress; sexual experience; substance use; treatment for violence-related injuries; exposure to secondhand smoke at home, school, and public places; ease of the purchase of cigarettes; the age at the first smoking experience; the average number of days they smoked in the last 30 days; and smoking cessation attempts [8,14,18,23]. The family’s perceived economic status was measured on a 5-point scale from “very high” to “very low”. Perceived health status was measured on a 5-point scale from “very healthy” to “very unhealthy”. Stress was measured on 5-point scale as the degree to which a participant felt stressed in a common day, ranging from “very low” to “very high”. Regarding sexual experience and treatment for violence-related injuries, data were collected from the KYRWBS based on the presence or absence of corresponding experiences in the last 12 months. Data on the exposure to secondhand smoke at home, school, and public places were obtained from the KYRWBS based on the experience of the last 7 days. As for the ease of the purchase of cigarettes in the last 30 days, the initial responses were “It was impossible to buy cigarettes”, “I could buy cigarettes with a lot of effort”, “I could buy cigarettes with a little effort”, or “I could easily buy cigarettes without effort”. These data were recategorized into “Impossible”, “Possible with efforts”, and “Easily possible”. For the age at the first smoking experience, data were assessed on a 13-point scale from “before entering elementary school” to “third year of high school” and recategorized as “under 13 years of age” and “over 13 years of age”, based on the national average age of the first smoking experience (13 years) [28]. The average number of smoking days in the last month was measured as “1–2 days per month”, “3–5 days per month”, “6–9 days per month”, “10–19 days per month”, “20–29 days per month”, and “every day”. Smoking cessation attempts were assessed based on whether or not there were any attempts to quit smoking in the last 12 months.

### 2.3. Statistical Analysis

As the survey data were extracted using a complex sample design, stratification variables, clusters, and weights were included in the analysis, and the finite population correction coefficient and equiprobability sampling without replacement were used as the standard error estimation method. SPSS Statistics 25.0 software (IBM Corp., Armonk, NY, USA) was used for the analysis.

The difference in correction variables according to the type of tobacco used by survey year was analyzed based on the complex sample general linear model and the Rao–Scott chi-square test. In addition, univariate and multivariate logistic regression analyses were used to determine the effect of the adolescents’ type of residence on the type of tobacco used. The significance level was set to less than 0.05.

## 3. Results

### 3.1. Participants’ Characteristics by Tobacco Type

The analysis of the participants’ characteristics for each type of tobacco for both survey years is described below.

In 2019, the most common residence type was “living with family or relatives” (96.3%), followed by “living in a dormitory or orphanage” (4.3%), and “living alone or in a boarding house” (2.4%). An analysis of the participants’ characteristics by tobacco type revealed significant differences between all variables (*p* < 0.001; Table 1).

The dual use group was the oldest, with an average age of 16.11 years. Male adolescents accounted for the highest proportion in all three groups based on tobacco type, particularly in the dual use group (78.2%). Regarding the perceived family’s economic status, both the e-cigarette and dual use groups reported the highest proportion of participants in the “very low” level (6.4%), and the e-cigarette group reported the highest proportion in the “very high” level (16.4%). Regarding the perceived health status, among the three groups, the highest percentage of participants reported a “very unhealthy” and “very healthy” status in the e-cigarette group (2.7% and 34.6%, respectively). As for stress, the e-cigarette group (7.9%) and the dual use group (19.9%) reported the highest percentage of participants with “very low” and “very high” levels of stress, respectively. The dual use group had the highest rate of sexual experience (43.1%) and the e-cigarette group had the highest rate of drug use (18.8%). The rate of treatment for violence-related injuries was the highest in the e-cigarette group (26.1%). Secondhand smoke exposure at home and school was the highest in the e-cigarette group (49.9% and 44.3%, respectively), while secondhand smoke exposure in public places was highest in the dual use group (69.8%). Regarding the ease of purchasing cigarettes, the most frequent response in the dual use group was “easy” (47.5%), while “difficult” and “impossible” were the most frequent responses in the cigarette group (46.9% and 20.0%, respectively). The highest rate for the first smoking experience under the age of 13 was reported by the e-cigarette group (27.6%). Furthermore, the largest proportion of cigarette users had an average number of 30 smoking days in the last 30 days (35.3%), while the highest percentage of the e-cigarette group (46.9%) and the dual use group (39.4%) had 1–2 days. Attempts to quit smoking were highest in the cigarette group (69.3%).

In 2020, the most common type of residence was living with family or relatives, reported by 2390 (93.1%) participants, followed by living in a dormitory or orphanage (128; 4.6%), and living alone or in a boarding house (57; 2.3%). Regarding the characteristics of the participants by tobacco type/form, stress was the only variable that was not significantly different (*p* = 0.598; Table 2).

The cigarette use group was the oldest, with an average age of 16.35 years. The proportion of male adolescents was the highest among all types of tobacco users, particularly in the dual use group (74.4%) compared with the other groups (*p* < 0.001). As for the perceived family’s economic status, “very low” and “very high” were reported by the highest proportion of participants in the dual use group (6.7% and 14.7%, respectively). The proportion of participants who reported a “healthy” perceived health status was the highest in the cigarette group and the dual use group, while the e-cigarette group (39.6%) had the highest percentage of participants with a ”very healthy” status. A “very unhealthy” lifestyle was reported by the highest proportion of participants in the dual use group (2.6%). “very low” and “very high” levels of stress were reported by the highest percentage of participants in the e-cigarette group (4.8%) and the dual use group (16.9%), respectively. The dual use group reported the highest rate of sexual experiences (43.3%), while the e-cigarette group reported the highest rate of substance use (3.9%). The rate of treatment for violence-related injuries was the highest in the e-cigarette group (95.2%). An exposure to secondhand smoke at home and school was highest in the e-cigarette group (37.7% and 24.7%, respectively). An exposure to secondhand smoke in public places was highest in the dual use group (64.0%). Regarding the ease of purchasing cigarettes, “easy” was the most frequent response in the dual use group (50.9%). However, responses of “difficult” and “impossible” were the highest in the e-cigarette group (49.8%) and cigarette group (16.7%), respectively. The first experience of smoking under the age of 13 was the highest in the e-cigarette group (19.7%). In addition, the highest proportion of participants in the cigarette group had an average of 30 smoking days in the last 30 days (46.5%), while the e-cigarette group (36.3%) and the dual use group (42.9%) mostly had 1–2 days. The number of attempts to quit smoking was highest in the cigarette group (70.1%).

### 3.2. Impact of Participants’ Type of Residence on Tobacco Type during the COVID-19 Pandemic

To determine the relationship between the type of residence and type of tobacco during the COVID-19 pandemic, data from the pre-pandemic (2019) and pandemic period (2020) were analyzed. The results are as follows.

The results of the analysis of data from 2019 are shown in Table 3. In the univariate logistic regression analysis, where only the type of residence was taken as the independent variable, the risk of using e-cigarettes was higher in the group living alone or living in a boarding house than in the group living with family or relatives (OR = 4.59, 95%CI = 2.64–7.97). However, in the multivariate logistic analysis that corrected the variables affecting the type of tobacco, the type of residence had no significant effect on the type of tobacco used (Table 3).

The results of the data from 2020 are shown in Table 4. In the univariate analysis, in which the type of residence was the only independent variable, the group living alone or living in a boarding house had a higher risk of using e-cigarettes (OR = 9.08, 95% CI [4.07–20.25]) and of dual use (OR = 3.70, 95%CI [1.81–7.54]) rather than cigarettes, compared with the group living with family or relatives. In addition, living in a dormitory or orphanage, compared with living with family or relatives, was associated with a higher risk of e-cigarette use (OR = 2.52, 95%CI [1.42–4.45]) and dual use (OR = 1.95, 95%CI [1.27–2.99]). In the multivariate logistic analysis that corrected the variables influencing the type of tobacco, the group living alone or living in a boarding house had a higher risk of using e-cigarettes (OR = 6.49, 95%CI [2.06–20.45]) rather than cigarettes compared with the group living with family or relatives. In addition, living in a dormitory or orphanage was associated with a higher risk of dual use than cigarette use (OR = 2.09, 95%CI [1.13–3.84]).

## 4. Discussion

The number of adolescent smokers in this study was lower during the COVID-19 pandemic (2020) than in the pre-COVID-19 period (2019). These findings are consistent with a number of studies analyzing changes in adolescent smoking rates before and during the COVID-19 pandemic [16]. Adolescents usually smoke outside the home in the absence of parents and family or while meeting with peers [17]. However, the shutdowns and social distancing measures imposed due to COVID-19 resulted in reduced smoking rates in adolescents due to an increased amount of time spent at home and difficulty in getting together with peers [16]. Thus, the results of this study could be attributable to the fact that the largest proportion of participants lived with their families and spent an increased amount of time at home due to social distancing, leading to a reduction in opportunities to smoke.

In this study, the pre-COVID-19 analysis showed that the relationship between the type of residence and the type of tobacco used by adolescent smokers was not significant; however, the data collected during the COVID-19 pandemic showed a significant relationship. A previous study showed that parental surveillance of adolescent smoking was significantly associated with the use of cigarettes, but not with e-cigarette use, as it is difficult for parents to notice smoking behavior in the case of e-cigarettes [29]; the lack of a significant relationship between the type of residence and type of tobacco in this study in the pre-COVID-19 analysis may be due to these factors. Although parental surveillance reduces adolescent smoking, it may not be appropriate to describe the characteristics of cohabitants and the residence type from the same perspective because the majority of adolescent smoking occurs outside the home [17], and the time they spend at home decreases [22]. In addition, previous studies used non-smokers as a reference group to identify the risk of the type of tobacco used [22,29,30], whereas this study only included adolescents who smoked and considered cigarettes users as the reference group. Taken together, adolescents spent more time outside the home than inside before the COVID-19 pandemic. Compared to cigarettes, the use of e-cigarettes is relatively easier for sole and dual users in terms of regulations in non-smoking zones [12].

This study found a significant relationship between the type of tobacco used by adolescents and the type of residence during the COVID-19 pandemic. Specifically, adolescents living alone or living in a boarding house showed a higher risk of using e-cigarettes than adolescents living with family members or relatives. Additionally, the risk of dual use was higher in adolescents living in dormitories or orphanages (social welfare facilities) than in those living with family members or relatives. The relationship between the type of residence and type of tobacco used may have been influenced by the following factors.

The results of this study showed that adolescents who were living alone or living in a boarding house had the highest risk of using e-cigarettes alone compared to other types of residence. Students living with their families or living in dormitories needed to follow the respective norms or rules, whereas, those living alone or living in a boarding house are independent of such discipline [31]. In addition to cohabitation with family members, social relationships and the sharing of physical spaces in the residence affects adolescent smoking, including e-cigarettes, because these factors serve to survey and control adolescent smoking [32]. It has been shown that adolescents considered using e-cigarettes less or quitting smoking because of the difficulty of visiting retailers or receiving deliveries as their parents were more likely to be home [8], while adolescents who were living alone or living in a boarding house were believed to be free from the surveillance and control of their cohabitants. In addition, it is highly difficult to perform surveillance on the purchase of e-cigarettes because, in addition to purchases through retail stores, which is the traditional method of purchasing cigarettes, e-cigarettes are purchased online or through friends [8]. The following environmental factors may also promote the use of e-cigarettes among adolescents: the explosive increase in online e-cigarette retailers in 2020 [3], the advertisement of e-cigarettes as a means of relieving boredom when spending time at home due to COVID-19 [33], and the choice of a variety of fragrances and designs based on their own preferences, making it a kind of symbolic product [4].

The risk of dual use in adolescents living in dormitories or orphanages (social welfare facilities) was higher than that in those living with family members or relatives. Stronger regulations for smoking in residential spaces reduces adolescent smoking [22]. However, it is not difficult to use e-cigarettes in public because they do not produce visible smoke, which is the main reason why smokers choose e-cigarettes [12]. Adolescents smokers are also well aware that it is difficult for others to monitor e-cigarettes because they differ in appearance from typical cigarettes; therefore, they often choose e-cigarettes for smoking in classrooms or public places [4]. Since there are no studies on the use of e-cigarettes among adolescents living in group housing, it is difficult to compare them directly. However, a study of college students showed that monitoring the smell and smoke of flammable cigarettes is used as a means of social control, which has an impact on reducing smoking [34]. In most shared residences, non-smoking regulations are imposed around cigarettes [25]. A study showed that a significant proportion of students living in dormitories used e-cigarettes, which influenced the choice of smokers or non-smokers living with them to use e-cigarettes [35]. In addition, adolescents tend to buy cigarettes in advance to prepare for unexpected situations because the purchase of cigarettes is not legal [8], and such uncertainties in purchase opportunity may have manifested in the dual use of cigarettes and e-cigarettes. Taken together, adolescents in dormitories or orphanages (social welfare facilities) are at an increased risk of dual use due to the combined effects of the aforementioned regulations and surveillance of institutions, social control and peer pressure, and uncertainty in the purchase of cigarettes. E-cigarette users are less aware of the need to include e-cigarettes in prohibiting smoking in communal living spaces than non-e-cigarette users [25]. Therefore, it is necessary to inform e-cigarette users of the risk of secondary smoking in a communal living space. A study of college students living in dormitories found that e-cigarettes are influenced by social norms, so it is necessary to monitor the co-residents’ use of e-cigarettes among each other and inform the co-residents’ perception of non-smoking areas to e-cigarette users. Thus, efforts should be made to provide information on the characteristics of new cigarettes in shared residential facilities where young people reside, educate them about the harm caused by the use of e-cigarettes, and formulate regulations for smoking cessation, including the use of e-cigarettes, in shared residential facilities.

Although this study did not directly measure the factors related to the type of residence and had the usual limitations of a cross-sectional study, it showed that changes related to the COVID-19 pandemic affected smoking behavior and the type of tobacco used. While this study is significant in identifying the relationship between the type of residence and the type of tobacco used by adolescents in the context of the COVID-19 pandemic, it has the following limitations. First, although this study explained that the type of residence included the characteristics of cohabitants and the spatial aspect of shared residential facilities, it did not include variables that directly measured the family’s surveillance and attention to adolescents, adolescents’ relationship with their cohabitants, quantitative measurement of the time spent in the residence, cigarette use in the residence, and the mode of cigarette purchase. Thus, future studies should investigate and analyze various variables related to the type of residence. Second, factors other than the correction variables used in this study, such as the composition of cohabitants, type of cigarette used, and type of cigarette used by friends, may influence smoking behavior. Third, since this study analyzed cross-sectional survey data, it was not possible to examine changes in the type of tobacco used by adolescents before and during the COVID-19 pandemic. Thus, although the study explains the difference in the types of tobacco used according to the type of residence before and during the pandemic, it cannot explain changes in the type of tobacco used.

## 5. Conclusions

Although the COVID-19 pandemic was expected to reduce smoking among adolescents, the use of e-cigarettes by adolescents has continued to increase. Changes in the type of residence and an increase in the time spent at home, due to shutdowns and remote classes implemented to prevent the spread of COVID-19, has affected adolescent smoking. This study compared data before and during the COVID-19 pandemic to investigate the relationship between the type of residence and tobacco used among adolescent smokers. The results showed that adolescents living in dormitories or orphanages (social services) had a higher risk of dual use than those living with family members or relatives, whereas the use of e-cigarettes alone was higher among adolescents living alone or living in a boarding house compared with those living with family members or relatives. Thus, various factors related to the type of residence had a combined effect on smoking behavior and the type of tobacco used. Therefore, it is important to pay attention to the characteristics of the type of residence that affects adolescent smoking in situations where social isolation is required, such as the outbreak of a novel infectious disease.

## Figures and Tables

**Table 1:** Characteristics of participants by tobacco type/form before the COVID-19 pandemic (2019). (*n* = 3774, *N* = 183,678).

Variable		Total (*n* = 3774, *N* = 183,678)		Cigarette (*n* = 1817, *N* = 86,688)		E-Cigarette (*n* = 298, *N* = 15,060)		Dual Use (*n* = 1659, *N* = 81,930)		t or F *	*p*
		*n* (*N*%) or M(SD)		*n* (*N*%) or M(SE)		*n* (*N*%) or M(SE)		*n* (*N*%) or M(SE)			
Age	(year)	15.97	(0.04)	15.92	(0.04)	15.49	(0.11)	16.11	(0.04)	365.98	<0.001
Sex	Male	1070	(27.1%)	1191	(33.4%)	236	(33.4%)	1277	(21.8%)	67.67	<0.001
	Female	2704	(72.9%)	626	(66.6%)	62	(66.6%)	382	(78.2%)		
Perceived family’s economic status	Very low	214	(5.2%)	80	(3.9%)	21	(6.4%)	113	(6.4%)	53.96	<0.001
	Low	565	(14.1%)	285	(14.4%)	44	(15.1%)	236	(13.6%)		
	Middle	1632	(44.1%)	846	(47.4%)	107	(36.9%)	679	(42.0%)		
	High	884	(23.7%)	436	(24.7%)	75	(25.3%)	373	(22.2%)		
	Very high	479	(12.9%)	170	(9.5%)	51	(16.4%)	258	(15.8%)		
Perceived health status	Very unhealthy	66	(1.8%)	20	(1.2%)	8	(2.7%)	38	(2.2%)	38.31	<0.001
	Unhealthy	314	(8.3%)	158	(8.6%)	24	(8.2%)	132	(7.9%)		
	Average	847	(22.0%)	420	(23.1%)	58	(18.2%)	369	(21.7%)		
	Healthy	1409	(38.1%)	736	(41.3%)	103	(36.3%)	570	(35.0%)		
	Very healthy	1138	(29.9%)	483	(25.8%)	105	(34.6%)	550	(33.3%)		
Stress	Very low	151	(3.9%)	43	(2.3%)	26	(7.9%)	82	(4.8%)	44.42	<0.001
	Low	495	(13.5%)	236	(13.5%)	54	(18.1%)	205	(12.7%)		
	Moderate	1301	(34.1%)	634	(34.1%)	94	(32.8%)	573	(34.2%)		
	High	1090	(29.3%)	561	(31.5%)	65	(21.6%)	464	(28.4%)		
	Very high	737	(19.2%)	343	(18.6%)	59	(19.6%)	335	(19.9%)		
Sexual intercourse	No	2505	(66.3%)	1350	(74.3%)	216	(71.4%)	939	(56.9%)	120.21	<0.001
	Yes	1269	(33.7%)	467	(25.7%)	82	(28.6%)	720	(43.1%)		
Substance use	No	3546	(93.9%)	1772	(97.7%)	242	(81.2%)	1532	(92.2%)	138.81	<0.001
	Yes	228	(6.1%)	45	(2.3%)	56	(18.8%)	127	(7.8%)		
Treatment for violence-related injuries	No	3421	(90.6%)	1729	(95.2%)	220	(73.9%)	1472	(88.7%)	152.64	<0.001
	Yes	353	(9.4%)	88	(4.8%)	78	(26.1%)	187	(11.3%)		
Exposure to secondhand smoke at home	No	2103	(56.3%)	1066	(59.6%)	147	(50.1%)	890	(53.8%)	17.13	<0.001
	Yes	1671	(43.7%)	751	(40.4%)	151	(49.9%)	769	(46.2%)		
Exposure to secondhand smoke at school	No	2404	(63.2%)	1266	(69.2%)	162	(55.7%)	976	(58.1%)	53.58	<0.001
	Yes	1370	(36.8%)	551	(30.8%)	136	(44.3%)	683	(41.9%)		
Exposure to secondhand smoke at public places	No	1318	(34.4%)	698	(37.3%)	120	(41.2%)	500	(30.2%)	25.84	<0.001
	Yes	2456	(65.6%)	1119	(62.7%)	178	(58.8%)	1159	(69.8%)		
Ease of cigarette purchase	Easy	998	(41.7%)	323	(33.1%)	65	(46.6%)	610	(47.5%)	56.59	<0.001
	Difficult	1053	(42.8%)	465	(46.9%)	59	(42.8%)	529	(39.8%)		
	Impossible	376	(15.4%)	203	(20.0%)	16	(10.7%)	157	(12.6%)		
Initiation of smoking before 13	No	3227	(86.0%)	1664	(92.0%)	215	(72.3%)	1348	(82.2%)	127.86	<0.001
	Yes	547	(14.0%)	153	(8.0%)	83	(27.6%)	311	(17.8%)		
Average number of smoking days per month	1~2	1309	(34.3%)	508	(27.3%)	141	(46.9%)	660	(39.4%)	2281.67	<0.001
	3~5	593	(15.2%)	211	(11.0%)	48	(15.6%)	334	(19.7%)		
	6~9	350	(9.5%)	130	(7.0%)	39	(12.7%)	181	(11.5%)		
	10~19	351	(9.4%)	186	(10.6%)	24	(9.5%)	141	(8.1%)		
	20~29	250	(6.7%)	154	(8.8%)	11	(4.1%)	85	(5.1%)		
	30	921	(24.8%)	628	(35.3%)	35	(11.2%)	258	(16.1%)		
Smoking cessation attempts	No	1189	(31.7%)	553	(30.7%)	129	(42.0%)	507	(30.8%)	4195.22	<0.001
	Yes	2585	(68.3%)	1264	(69.3%)	169	(58.0%)	1152	(69.2%)		
The type of living residence	Living with family or relative	3496	(93.3%)	1711	(94.6%)	258	(86.6%)	1527	(93.2%)	46.75	<0.001
	Living alone or living in a boarding house	94	(2.4%)	32	(1.8%)	24	(7.7%)	38	(2.0%)		
	Living in a dormitory or orphanage	184	(4.3%)	74	(3.5%)	16	(5.7%)	94	(4.8%)		

*n* = unweight sample size; *N* = weight sample size; *N*% = weighted %; M = mean; SD = standard deviation; * calculated by Rao–Scott *x*^2^ test.

**Table 2:** Characteristics of participants by tobacco type/form during the COVID-19 pandemic (2020). (*n*= 2575, *N* = 122,347).

Variable		Total (*n* = 2575, *N* = 122,347)		Cigarette (*n* = 1407, *N* = 66,716)		E-Cigarette (*n* = 205, *N* = 10,808)		Dual Use (*n* = 965, *N* = 44,823)		t or F *	*p*
		*n* (*N*%) or M (SD)		*n* (*N*%) or M (SD)		*n* (*N*%) or M (SD)		*n* (*N*%) or M (SD)			
Age	(year)	16.29	(0.03)	16.35	(0.05)	15.89	(0.11)	16.30	(0.05)	326.62	<0.001
Sex	Male	1803	(71.3%)	954	(68.8%)	150	(73.7%)	699	(74.4%)	9.42	0.009
	Female	772	(28.7%)	453	(31.2%)	55	(26.3%)	264	(25.6%)		
Perceived family’s economic status	Very low	307	(12.1%)	70	(4.6%)	10	(3.9%)	65	(6.7%)	22.14	0.006
	Low	641	(25.9%)	223	(15.5%)	24	(10.2%)	137	(13.3%)		
	Middle	1098	(42.4%)	620	(43.5%)	92	(46.8%)	386	(39.7%)		
	High	641	(25.9%)	347	(25.7%)	59	(28.7%)	235	(25.5%)		
	Very high	307	(12.1%)	147	(10.7%)	20	(10.5%)	140	(14.7%)		
Perceived health status	Very unhealthy	32	(1.4%)	8	(0.8%)	1	(0.3%)	23	(2.6%)	33.15	<0.001
	Unhealthy	225	(9.1%)	122	(9.2%)	17	(9.0%)	86	(9.1%)		
	Average	599	(23.2%)	334	(23.7%)	50	(23.7%)	215	(22.5%)		
	Healthy	920	(35.4%)	530	(38.0%)	58	(27.4%)	332	(33.5%)		
	Very healthy	799	(30.8%)	413	(28.4%)	79	(39.6%)	307	(32.2%)		
Stress	Very low	99	(3.7%)	45	(3.1%)	12	(4.8%)	42	(4.3%)	6.68	0.598
	Low	372	(14.7%)	207	(14.3%)	34	(17.4%)	131	(14.6%)		
	Moderate	928	(35.7%)	513	(36.1%)	71	(35.3%)	344	(35.4%)		
	High	765	(30.1%)	427	(31.2%)	59	(28.0%)	279	(28.9%)		
	Very high	411	(15.8%)	215	(15.2%)	29	(14.6%)	167	(16.9%)		
Sexual intercourse	No	1694	(65.3%)	984	(69.9%)	148	(72.9%)	562	(56.7%)	49.94	<0.001
	Yes	881	(34.7%)	423	(30.1%)	57	(27.1%)	401	(43.3%)		
Substance use	No	2496	(97.1%)	1371	(97.8%)	199	(97.4%)	926	(96.1%)	5.93	0.043
	Yes	79	(2.9%)	36	(2.2%)	6	(2.6%)	37	(3.9%)		
Treatment for violence-related injuries	No	2426	(94.4%)	1366	(97.1%)	168	(82.8%)	892	(93.3%)	80.80	<0.001
	Yes	149	(5.6%)	41	(2.9%)	37	(17.2%)	71	(6.7%)		
Exposure to secondhand smoke at home	No	1756	(69.3%)	989	(71.1%)	125	(62.3%)	642	(68.2%)	7.84	0.033
	Yes	819	(30.7%)	418	(28.9%)	80	(37.7%)	321	(31.8%)		
Exposure to secondhand smoke at school	No	2106	(81.6%)	1219	(86.1%)	150	(75.3%)	737	(76.3%)	43.12	<0.001
	Yes	469	(18.4%)	188	(13.9%)	55	(24.7%)	226	(23.7%)		
Exposure to secondhand smoke at public places	No	1069	(41.4%)	644	(44.9%)	83	(42.7%)	342	(36.0%)	18.65	<0.001
	Yes	1506	(58.6%)	763	(55.1%)	122	(57.3%)	621	(64.0%)		
Ease of cigarette purchase	Easy	683	(43.7%)	301	(37.9%)	28	(38.8%)	354	(50.9%)	31.34	<0.001
	Difficult	663	(42.7%)	351	(45.4%)	35	(49.8%)	277	(38.8%)		
	Impossible	227	(13.6%)	142	(16.7%)	8	(11.4%)	77	(10.3%)		
Initiation of smoking before 12	No	2152	(83.6%)	1231	(87.4%)	162	(80.3%)	761	(79.0%)	43.42	<0.001
	Yes	421	(16.3%)	176	(12.6%)	43	(19.7%)	202	(21.0%)		
Average number of smoking days per month	1~2	792	(30.5%)	301	(21.2%)	73	(36.3%)	418	(42.9%)	3146.73	<0.001
	3~5	298	(11.1%)	110	(7.3%)	37	(18.3%)	151	(15.2%)		
	6~9	222	(8.0%)	89	(5.6%)	23	(10.0%)	110	(11.1%)		
	10~19	280	(11.6%)	146	(11.7%)	32	(14.7%)	102	(10.7%)		
	20~29	166	(6.4%)	114	(7.7%)	14	(7.4%)	38	(4.2%)		
	30	817	(32.3%)	647	(46.5%)	26	(13.2%)	144	(15.9%)		
Smoking cessation attempts	No	805	(31.8%)	415	(29.9%)	100	(47.4%)	290	(30.8%)	2855.01	<0.001
	Yes	1770	(68.2%)	992	(70.1%)	105	(52.6%)	673	(69.2%)		
The type of living residence	Living with family or relative	2390	(93.1%)	1344	(95.8%)	174	(85.3%)	872	(90.8%)	56.65	<0.001
	Living alone or living in a boarding house	57	(2.3%)	13	(0.9%)	14	(7.4%)	30	(3.2%)		
	Living in a dormitory or orphanage	128	(4.6%)	50	(3.2%)	17	(7.3%)	61	(6.0%)		

*n* = unweight sample size; *N* = weight sample size; *N*% = weighted %; M = mean; SD = standard deviation; * calculated by Rao–Scott *x*^2^ test.

**Table 3:** The impact of the type of residence of the participants before the COVID-19 pandemic (2019) on the type of tobacco.

Characteristics	Categories	Unadjusted (Ref. Cigarette)						Adjusted * (Ref. Cigarette)					
		E-Cigarette			Dual Use			E-Cigarette			Dual Use		
		OR	95% CI	*p*	OR	95% CI	*p*	OR	95% CI	*p*	OR	95% CI	*p*
The type of living residence	Living with family or relative	1						1					
	Living alone or living in a boarding house	4.59	(2.64–7.97)	<0.001	1.11	(0.66–1.88)	0.686	2.23	(0.95–5.25)	0.066	0.72	(0.34–1.56)	0.408
	Living in a dormitory or orphanage	1.77	(0.98–3.20)	0.059	1.39	(0.98–1.99)	0.067	0.81	(0.31–2.11)	0.665	0.75	(0.38–1.45)	0.385
		Nagelkerke R^2^ = 0.010, Cox and Snell R^2^ = 0.009						Nagelkerke R^2^ = 0.373, Cox and Snell R^2^ = 0.307					

Ref. = reference group; OR = odd ratio; CI = confidence interval; * adjusted age, sex, perceived family’s economic status, perceived health status, stress, sexual intercourse, substance use, treatment for violence-related injuries, exposure to secondhand smoke at home, exposure to secondhand smoke at school, exposure to secondhand smoke at public places, ease of cigarette purchase, initiation of smoking before 12, average number of smoking days per month, and smoking cessation attempt.

**Table 4:** Impact of the residence type of the participants on tobacco type during the COVID-19 pandemic (2020).

Characteristics	Categories	Unadjusted (Ref. Cigarette)						Adjusted * (Ref. Cigarette)					
		E-Cigarette			Dual Use			E-Cigarette			Dual Use		
		OR	95% CI	*p*	OR	95% CI	*p*	OR	95% CI	*p*	OR	95% CI	*p*
The type of living residence	Living with family or relative	1						1					
	Living alone or living in a boarding house	9.08	(4.07–20.25)	<0.001	3.70	(1.81–7.54)	0.000	6.49	(2.06–20.45)	0.001	2.64	(0.93–7.51)	0.068
	Living in a dormitory or orphanage	2.52	(1.42–4.45)	0.002	1.95	(1.27–2.99)	0.002	2.03	(0.83–4.92)	0.119	2.09	(1.13–3.84)	0.018
		Nagelkerke R^2^ = 0.023, Cox and Snell R^2^ = 0.019						Nagelkerke R^2^ = 0.400, Cox and Snell R^2^ = 0.325					

Ref. = reference group; OR = odd ratio; CI = confidence interval; * adjusted age, sex, perceived family’s economic status, perceived health status, stress, sexual intercourse, substance use, treatment for violence-related injuries, exposure to secondhand smoke at home, exposure to secondhand smoke at school, exposure to secondhand smoke at public places, ease of cigarette purchase, initiation of smoking before 12, average number of smoking days per month, and smoking cessation attempt.

## Data Availability

Raw data were provided by the KDCPA from the KYRBWS. Data were made available after obtaining permission. URL: https://www.kdca.go.kr/yhs/ (accessed on 26 April 2022).

## References

[1] Staff J., Kelly B.C., Maggs J.L., Vuolo M. (2022). Adolescent electronic cigarette use and tobacco smoking in the Millennium Cohort Study. Addiction.

[2] Hammond D., Reid J.L., Rynard V.L., Fong G.T., Cummings K.M., McNeill A., Hitchman S., Thrasher J.F., Goniewicz M.L., Bansal-Travers M. (2019). Prevalence of vaping and smoking among adolescents in Canada, England, and the United States: Repeat national cross sectional surveys. BMJ.

[3] Van der Eijk Y., Tan Ping Ping G., Ong S.E., Tan Li Xin G., Li D., Zhang D., Min Shuen L., Kee Seng C. (2021). E-Cigarette Markets and Policy Responses in Southeast Asia: A Scoping Review. Int. J. Health Policy Manag..

[4] Barrington-Trimis J.L., Leventhal A.M. (2018). Adolescents’ use of “pod mod” e-cigarettes—urgent concerns. New Engl. J. Med..

[5] Huizink A.C. (2022). Trends and associated risks in adolescent substance use: E-cigarette use and nitrous oxide use. Curr. Opin. Psychol..

[6] Bluestein M., Kelder S., Perry C.L., Pérez A. (2019). Exploring associations between the use of alcohol and marijuana with e-cigarette use in a USA nationally representative sample of young adults. Int. J. Health Sci..

[7] Rachel E.C., Brandenberger K.J., Battey-Muse C.M., Gardenhire D.S. (2022). 2021 Year in Review: E-Cigarettes, Hookah Use, and Vaping Lung Injuries During the COVID-19 Pandemic. Respir. Care.

[8] Gaiha S.M., Lempert L.K., Halpern-Felsher B. (2020). Underage Youth and Young Adult e-Cigarette Use and Access Before and During the Coronavirus Disease 2019 Pandemic. JAMA Netw. Open.

[9] Owotomo O., Maslowsky J., Loukas A. (2018). Perceptions of the Harm and Addictiveness of Conventional Cigarette Smoking Among Adolescent E-Cigarette Users. J. Adolesc. Health.

[10] Choi J.Y., Na K.I. (2022). A panel study for smokers and its in-depth analysis. Public Health Wkly. Rep..

[11] Gordon T., Karey E., Rebuli M.E., Escobar Y.H., Jaspers I., Chen L.C. (2022). E-Cigarette Toxicology. Annu. Rev. Pharm. Toxicol..

[12] Glantz S.A., Bareham D.W. (2018). E-Cigarettes: Use, Effects on Smoking, Risks, and Policy Implications. Annu. Rev. Public Health.

[13] Her W. (2020). Factors Influencing Type of Cigarette Smoked among Adolescents: Focusing on the Differences between Conventional Cigarette and Electronic Cigarette. Health Soc. Welf. Rev..

[14] Cho M.S. (2021). Factors Associated with Cigarette, E-Cigarette, and Dual Use among South Korean Adolescents. Healthcare.

[15] Bares C., Lopez-Quintero C. (2021). Shared Environmental Influences on Electronic Cigarette Use Among Adolescent and Young Adult Females. Nicotine Tob. Res. Off. J. Soc. Res. Nicotine Tob..

[16] Layman H.M., Thorisdottir I.E., Halldorsdottir T., Sigfusdottir I.D., Allegrante J.P., Kristjansson A.L. (2022). Substance Use Among Youth During the COVID-19 Pandemic: A Systematic Review. Curr. Psychiatry Rep..

[17] Trucco E.M., Cristello J.V., Sutherland M.T. (2021). Do parents still matter? The impact of parents and peers on adolescent electronic cigarette use. J. Adolesc. Health.

[18] Hanafin J., Sunday S., Clancy L. (2021). Friends and family matter Most: A trend analysis of increasing e-cigarette use among Irish teenagers and socio-demographic, personal, peer and familial associations. BMC Public Health.

[19] Kreslake J.M., Simard B.J., O’Connor K.M., Patel M., Vallone D.M., Hair E.C. (2021). E-Cigarette Use Among Youths and Young Adults During the COVID-19 Pandemic: United States, 2020. Am. J. Public Health.

[20] Thorlindsson T., Valdimarsdottir M., Hrafn Jonsson S. (2012). Community social structure, social capital and adolescent smoking: A multi-level analysis. Health Place.

[21] Fowler P.J., Henry D.B., Marcal K.E. (2015). Family and housing instability: Longitudinal impact on adolescent emotional and behavioral well-being. Soc. Sci. Res..

[22] Barrington-Trimis J.L., Berhane K., Unger J.B., Cruz T.B., Huh J., Leventhal A.M., Urman R., Wang K., Howland S., Gilreath T.D. (2015). Psychosocial Factors Associated With Adolescent Electronic Cigarette and Cigarette Use. Pediatrics.

[23] Wellman R.J., Sylvestre M.P., O’Loughlin E.K., Dutczak H., Montreuil A., Datta G.D., O’Loughlin J. (2018). Socioeconomic status is associated with the prevalence and co-occurrence of risk factors for cigarette smoking initiation during adolescence. Int. J. Public Health.

[24] Lee T.H., Kim W. (2021). Differences in electronic cigarette use among adolescents in Korea: A nationwide analysis. J. Subst Abus. Treat.

[25] Patel M., Donovan E.M., Liu M., Solomon-Maynard M., Schillo B.S. (2022). Policy Support for Smoke-Free and E-Cigarette Free Multiunit Housing. Am. J. Health Promot..

[26] Korea Disease Control and Prevention Agency (2022). 17th(2021) Korea Youth Risk Behavior Web-Based Survey Statistics.

[27] Sokolovsky A.W., Hertel A.W., Micalizzi L., White H.R., Hayes K.L., Jackson K.M. (2021). Preliminary impact of the COVID-19 pandemic on smoking and vaping in college students. Addict. Behav..

[28] Korea Disease Control and Prevention Agency (2022). 16th(2020) Korea Youth Risk Behavior Web-Based Survey Statistics.

[29] Fotiou A., Kanavou E., Stavrou M., Richardson C., Kokkevi A. (2015). Prevalence and correlates of electronic cigarette use among adolescents in Greece: A preliminary cross-sectional analysis of nationwide survey data. Addict. Behav..

[30] Moore G.F., Angel L., Gray L., Copeland L., van Godwin J., Segrott J., Hallingberg B. (2020). Associations of Socioeconomic Status, Parental Smoking and Parental E-Cigarette Use with 10-11-Year-Old Children’s Perceptions of Tobacco Cigarettes and E-Cigarettes: Cross Sectional Analysis of the CHETS Wales 3 Survey. Int. J. Env. Res. Public Health.

[31] Jeon H. (2017). The impact of on-campus residence on early institutional commitment among college freshmen: A focus on faculty and peer interactions. Korean Educ. Inq..

[32] Shih R.A., Parast L., Pedersen E.R., Troxel W.M., Tucker J.S., Miles J.N.V., Kraus L., D’Amico E.J. (2017). Individual, peer, and family factor modification of neighborhood-level effects on adolescent alcohol, cigarette, e-cigarette, and marijuana use. Drug Alcohol Depend..

[33] Ramamurthi D., Chau C., Jackler R.K. (2021). Exploitation of the COVID-19 pandemic by e-cigarette marketers. Tob. Control.

[34] Noland M., Ickes M.J., Rayens M.K., Butler K., Wiggins A.T., Hahn E.J. (2016). Social influences on use of cigarettes, e-cigarettes, and hookah by college students. J. Am. Coll Health.

[35] Doxbeck C.R., Osberg T.M. (2021). It’s Not All Smoke and Mirrors: The Role of Social Norms, Alcohol Use, and Pandemic Partying in e-Cigarette Use During COVID-19. Subst. Use Misuse.

